# The Impact of HIV, an Antiretroviral Programme and Tuberculosis on Mortality in South African Platinum Miners, 1992–2010

**DOI:** 10.1371/journal.pone.0038598

**Published:** 2012-06-22

**Authors:** Megan S. C. Lim, Robert J. Dowdeswell, Jill Murray, Nigel Field, Judith R. Glynn, Pam Sonnenberg

**Affiliations:** 1 Research Department of Infection and Population Health, University College London, London, United Kingdom; 2 Rustenburg Platinum Mines Limited, Rustenburg, South Africa; 3 National Institute for Occupational Health, National Health Laboratory Service and School of Public Health, University of the Witwatersrand, Johannesburg, South Africa; 4 Department of Infectious Disease Epidemiology, London School of Hygiene and Tropical Medicine, London, United Kingdom; Boston University, United States of America

## Abstract

**Background:**

HIV and tuberculosis (TB) are the most common causes of death in South Africa. Antiretroviral therapy (ART) programmes should have had an impact on mortality rates. This study describes the impact of HIV, a Wellness (HIV/ART) programme and TB on population-wide trends in mortality and causes of death among South African platinum miners, from before the HIV epidemic into the ART era.

**Methodology/Principal Findings:**

Retrospective analysis was conducted using routinely-collected data from an open cohort. Mortality and causes of death were determined from multiple sources, including cardiorespiratory autopsy records. All-cause and cause-specific mortality rates were calculated by calendar year. 41,665 male miners were observed for 311,938 person years (py) with 3863 deaths. The all-cause age-standardised mortality rate increased from 5.9/1000py in 1992 to 20.2/1000py in 2002. Following ART rollout in 2003, annual mortality rates fluctuated between 12.4/1000py and 19.3/1000py in the subsequent 7 years. Half of all deaths were HIV-related and 21% were caused by TB. Half (50%) of miners who died of HIV after ART rollout had never been registered on the Wellness programme. TB was the most common cause of death in HIV positive miners, increasing from 28% of deaths in the pre-ART period to 41% in the post-ART period.

**Conclusions/Significance:**

This population-based cohort experienced a rapid increase in mortality from 1996 to 2003 due to increases in HIV and TB mortality. Following ART rollout there was a decrease in mortality, but a steady decrease has not been sustained. Possible explanations for these trends include the changing composition of the workforce, maturation of the HIV epidemic, insufficient uptake of ART and an increase in the proportion of deaths due to TB. In order to make a significant and sustained reduction in mortality in this population, expanding and integrating HIV and TB care and treatment is essential.

## Introduction

While mortality rates worldwide have declined over the past two decades, there have been substantial increases in adult mortality in South Africa, mainly due to HIV and tuberculosis (TB). [1] Since the widespread implementation of antiretroviral therapy (ART) programmes, six published reports from sub-Saharan Africa on the impact of ART at a population level have demonstrated reduced mortality. [2]–[7] ART has not yet reached everyone in need, however, and HIV mortality rates are still unacceptably high. [8] ART has also been associated with reductions in TB incidence and mortality in South Africa, [9], [10] yet TB remains the most common cause of adult death in South Africa. [11] In 2008 it was estimated that 60% of those requiring ART in South Africa were not receiving it. [12] Likewise, an estimated 28% of TB cases went undetected in 2010 [13].

The HIV epidemic affects individuals in their productive working years of life. Surveys on mining operations in South Africa have reported HIV prevalences of 15% to 27%. [14], [15] TB rates in South African gold miners are among the highest in the world, and HIV/TB co-infection is common. [16] Many occupational settings provide healthcare for workers, which includes voluntary counselling and testing (VCT) services, care for HIV positive employees within a Wellness programme and, more recently, free ART [17], [18].

This study measures trends in mortality in a population of South African platinum miners from 1992 to 2010, covering the period before the HIV epidemic until several years after the rollout of ART. We estimate cause-specific mortality (using medical records and findings from autopsies of cardiorespiratory organs) to describe the impact of HIV, a Wellness programme that includes ART, and TB on mortality in this population.

## Methods

### Setting

The study was based on a platinum mine in Rustenburg, North West Province, South Africa, where HIV seroprevelance was estimated to be 24.6% in 2002. [14] Medical care is provided free of charge by the company and includes primary care and hospital facilities. All miners join a provident fund which provides a death benefit payable to the employee’s next-of-kin in the event of death within 12 months of being “medically boarded” (i.e. retired on the grounds of ill-health). The company’s HIV programme includes prevention, VCT, and a “Wellness” programme for HIV-positive employees, through which miners could access ART since April 2003. [17], [19] Employees need to be registered on Wellness to monitor their CD4 counts, clinical status and, where appropriate, to receive free ART provided by the mining company. During the period of the study, patients were eligible for ART if they had a CD4 count of <250 cells/ml, WHO stage 4, or WHO stage 3 with a CD4 count of <350 cells/ml. A TB service, which includes screening, diagnostic tests and treatment, is provided free of charge and miners diagnosed with TB receive treatment in accordance with South African National guidelines, through a dedicated TB clinic.

### Data Collection

The study utilised a number of routinely-collected retrospective databases, with individuals linked by a unique company number. These included a personnel database with details of employment dates; medical databases (hospital death register, Wellness programme, TB clinic, VCT, hospital laboratory); provident fund records; and the National Institute for Occupational Health (NIOH) autopsy database, which has included data on cause of death in a standardised format since 1996. Autopsies of the cardio-respiratory organs are conducted on miners for compensation purposes, with the consent of next of kin and regardless of the clinical cause of death.

### Defining the Cohort

Personnel records were used to construct an open cohort of all semiskilled and unskilled male miners (comprising underground miners, labourers and surface plant workers). Skilled workers were excluded as they do not use the mine medical services. Subjects entered the cohort on 1 January 1992 or on the date of employment if later than this. They remained in the cohort until they died, left the mine or until 31 December 2010. The date of censoring for medically boarded employees was extended by 12 months (or until death, or 31 December 2010 if this occurred earlier) as these employees remained at risk and under observation while eligible for the death benefit. We excluded 3792 females since they comprised a small proportion of the workforce and their risk behaviours, health service utilisation, and background mortality rates can differ substantially from males in this manual labour employment setting. Furthermore, in the early study period, very few women were employed on the mines making it difficult to explore time trends.

### Determination of Cause of Death and HIV Status at Death

The most likely cause of death was determined using all available data sources. If multiple causes of death were plausible, the main cause was determined by the clinical judgement of three clinicians (including one pathologist), with autopsy findings taking precedence. For example, a TB death would be one where TB was identified clinically and/or on autopsy. However, if the autopsy identified another cause of death then this case would not be categorised as a TB death despite a clinical diagnosis of TB.

Men were classified as HIV positive if they had a recorded HIV positive test; if they were identified as HIV positive through the ART or TB treatment databases; if their medical record or death certificate stated HIV, AIDS or if a euphemism such as ‘immune compromised’; or if their cause of death was a clearly AIDS-related condition (*Pneumocystis jirovecii* pneumonia (PCP), aspergillus, nocardia pneumonia, cryptococcal disease, oesophageal candidiasis, lymphadenopathy, interstitial pneumonitis, cytomegalovirus pneumonia, toxoplasmosis, or Kaposi’s sarcoma). All men who were not known to be positive were classified as ‘HIV negative or unknown.’

Causes of death were classified into one of four Global Burden of Disease groups, as described by Bradshaw, et. al.: I) communicable, II) non-communicable, III) violence and injuries, and IV) HIV-related. [20] The cause of death was considered to be HIV-related if the miner was HIV positive and the cause of death was likely to be related to HIV (e.g. excluding violence or injury, cardiovascular disease, most malignancies). For example, TB or bacterial pneumonia in an HIV-positive individual would be included in HIV/AIDS-related deaths, whereas, in an HIV-negative or HIV-unknown individual, these would be included in the communicable diseases group.

### Analysis

Data were entered and cleaned, and personal identifiers were removed. Records were expanded into person years at risk (py) by calendar year (1992 to 2010) and 10-year age categories. Median current age was calculated for each year as the man’s age on January 1^st^ of that year, or on entry to cohort if later. All-cause and cause-specific mortality rates were calculated by calendar year. Direct age standardisation was applied, using the average age distribution for the entire cohort in 10-year age groups as the standard. Age-adjusted Poisson regression coefficients determined the average change in rate per year during the study period since 1992. Analyses were conducted using Stata 10.

### Ethics Statement

Ethical approval was obtained from the Human Research Ethics Committee (Medical) of the University of the Witwatersrand, South Africa (M041028), and the UCL Research Ethics Committee, United Kingdom (1641/001). The Committees specifically addressed issues of consent and confidentiality. Given the retrospective nature of the study; high mortality; use of routine databases linked by a unique number; and removal of personal identifiers prior to analysis, specific written consent for inclusion in this research was not required. Autopsies were conducted with written consent from the next of kin, in accordance with the South African Occupational Diseases in Mines and Works Act. The project is covered by the UCL Data Protection Registration (Reference No Z6364106/2008/8/18, Section 19, Research: Health Research).

## Results

The cohort of 41,665 miners was observed for 311,938 person years (Table 1). There were fluctuations in the median size, age and duration of employment of the workforce over the period, including two periods (2005 and 2009) when a number of workers accepted voluntary retrenchment packages. The median age of the cohort was 35.8 years in 1992, reaching 45.3 years by 2005 and then reducing to 40.9 years by 2010. There were 3863 deaths from 1992 to 2010, giving a crude mortality rate of 12.4/1000py (95%CI 12.0–12.8). Twelve percent of deaths (n = 450) were in those who had been medically boarded. If medically boarded men were censored when they left employment, the overall mortality rate was 11.0/1000py. Mortality increased with age group: from 5.0/1000py in those aged <25 years, to 6.7, 10.8, 17.4 and 22.6 in those aged 25–34, 35–44, 45–54 and 55+ years, respectively. Results from autopsy of cardiorespiratory organs were available for 36% (n = 1401) of deaths.

**Table 1:** Description of cohort.

Year	Total Cohort size	Number of men entering	Number of men exiting						Person years at risk	Median current age	Median age new employees	Median age exiting employees	Median years in service	Number new Wellness registrations	Number commencing ART	Median (IQR) CD4 count in those commencing ART^3^
			Death	Medically boarded^1^	Resigned	Dismissed	Other^2^	Total								
**1992**	20428	1408	94	39	655	402	98	1249	19249	35.8	32.2	35.4	6.8			
**1993**	20359	1175	111	57	663	351	87	1249	18981	36.7	32.9	35.7	7.7			
**1994**	20332	1214	118	35	529	243	69	1012	19178	37.4	29.9	36.0	8.4			
**1995**	20302	983	115	38	570	704	112	1529	19248	38.2	30.8	38.8	9.2			
**1996**	20036	1264	121	66	627	497	114	1390	18722	38.8	29.2	39.6	10.1			
**1997**	19226	569	149	62	487	240	20	957	18484	39.6	28.7	39.2	11.1			
**1998**	18702	431	158	61	437	728	7	1392	17782	40.5	29.0	46.5	12.1			
**1999**	18085	782	177	27	247	408	168	1060	17124	41.0	31.6	41.6	12.9			
**2000**	17518	491	268	115	628	580	157	1658	16471	41.8	30.8	41.1	13.8			
**2001**	16426	563	288	137	438	386	11	1190	15451	42.7	26.8	42.8	14.5			
**2002**	16440	1203	319	201	497	342	12	1236	15202	43.2	30.9	44.0	15.0			
**2003**	16665	1464	325	188	410	325	147	1320	15422	43.5	31.8	43.1	15.3	274	204	140 (72–218)
**2004**	15533	185	265	222	593	290	15	1287	14847	44.5	31.1	45.8	16.3	617	401	161 (87–250)
**2005**	14434	187	216	285	2230	166	15	2822	12533	45.3	29.3	48.7	17.3	452	316	163 (92–243)
**2006**	13656	2049	159	101	461	132	22	1024	11673	44.5	32.6	48.1	12.4	522	330	167 (95–242)
**2007**	15314	2677	211	116	478	157	37	971	13972	43.6	32.3	44.5	8.5	444	161	184 (98–239)
**2008**	18689	4348	242	98	534	213	21	1114	15660	41.9	32.5	44.8	4.4	416	113	162 (82–232)
**2009**	18281	704	315	119	1714	285	223	2616	16875	41.9	29.7	49.5	2.9	498	318	198 (95–270)
**2010**	16588	924	212	68	979	193	72	1598	15070	40.9	33.8	48.5	3.1	378	235	
**TOTAL**	**41665**	**22621**	**3863**	**2035**	**13177**	**6642**	**1407**	**26674**	**311938**	**40.7**	**31.5**	**43.4**	**10.2**	**3601**	**2078**	**162 (89–240)**

1 Includes those who later died (n = 450), these men are counted in both the death and medically-boarded columns.

2 Predominantly transfers to another mine or unknown reasons.

3 Only available for 1312 (63%) patients.

### Mortality Trends and Causes

**Figure 1:**
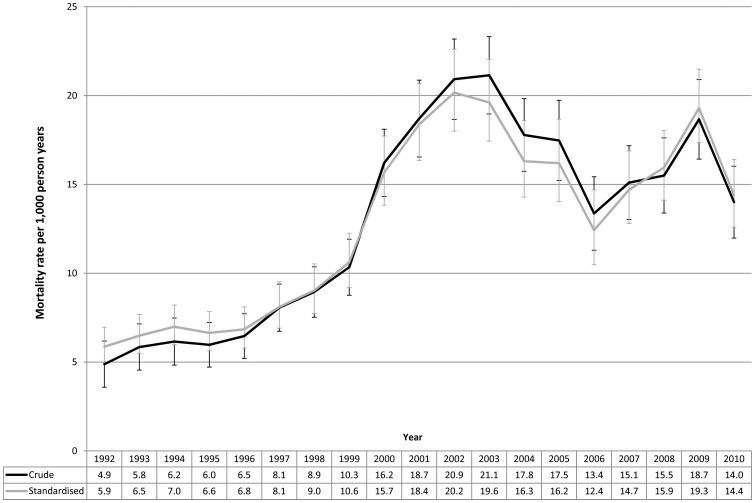
Crude and age standardised mortality rates (all-cause) in cohort, by year, 95% error bars shown.

Figure 1 shows the crude and age-standardised mortality rate over time. The all-cause crude mortality rate increased rapidly from a baseline of 4.9/1000py in 1992 and peaked at 21.1/1000py in 2003. The mortality rate fluctuated but remained high between 2003 and 2010. Age-standardised mortality followed a similar pattern, rising from 5.9/1000py in 1992 to 20.2/1000py in 2002, and fluctuating in the following years.

**Figure 2:**
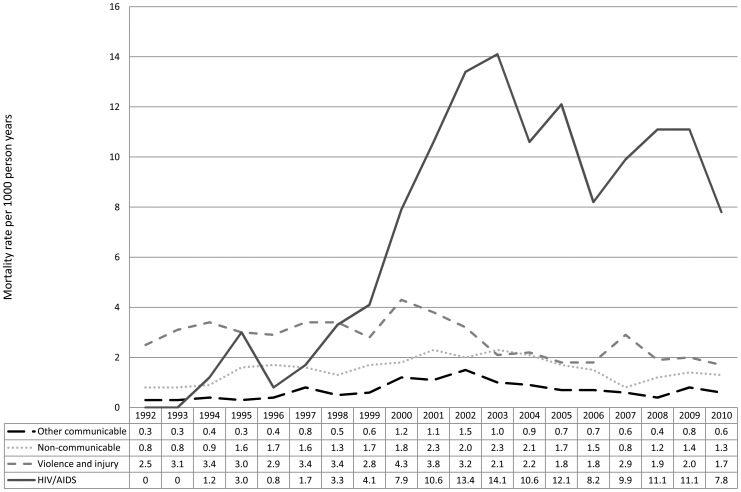
Cause-specific mortality rates, by year.

Figure 2 shows mortality rates over the period 1992 to 2010, in the four Burden of Disease groups. There were no known HIV/AIDS-related deaths prior to 1994, with HIV/AIDS-related mortality increasing rapidly from the mid-1990s, peaking at 14.1/1000py in 2003, dropping to 8.2/1000py by 2006 and fluctuating in the subsequent four years. Mortality rates remained stable between 1992 and 2010 for communicable diseases other than HIV (average change of +2% per year, 95%CI −1%, +4%) and for non-communicable diseases (−1% per year, 95%CI −2%, +1%). Rates of death from violence and injuries declined by an average of 3% (95%CI −4%,−2%) per year over the study period. TB mortality increased from 0.2/1000py in 1992 to a peak of 6.7/1000py in 2003, and fluctuating in subsequent years; an overall increase between 1992 and 2010 of 14% per year (95%CI +12%, +16%).

Table 2 shows cause of death by HIV status, Wellness programme registration and time period. Of the 3863 deaths, 78% were from natural causes. Overall, 50% of deaths were HIV/AIDS-related and 21% were due to TB. In HIV positive miners, the proportion of deaths attributed to TB increased significantly over time, from 28% in the pre-ART period to 41% in the post-ART period (p<0.01). There were no other significant changes in the proportions of deaths from the different causes among HIV positive men before and after the rollout of ART. In HIV negative miners, the proportion of deaths due to TB was 5.4% in the pre-ART period and 6.2% in the post-ART period (p = 0.6).

**Table 2:** Cause of death by HIV status, time period, and enrolment in Wellness programme – number, rate/1000py, and proportion of deaths.

*Cause of death*	*ALL*			*HIV negative/unknown*		*HIV positive*					
						*Pre ART (1992–March 2003)*		*Post ART (April 2003–2010)*			
								*Never on Wellness programme*		*Registered on Wellness programme*	
	n	%	rate	n	%	n	%	n	%	n	%
**Total deaths**	**3863**	**100**	**12.4**	**1822**		**778**		**633**		**630**	
Natural	2994	78	9.6	1015	56	750	96	616	97	613	97
Violence and injuries^1^	869	22	2.8	807	44	28	4	17	3	17	3
**Communicable**											
Overall	1996	52	6.4	204	11	679	87	555	88	558	89
Tuberculosis, any site	814	21	2.6	82	5	219	28	270	43	243	39
PCP	96	2	0.3	0	0	41	5	31	5	24	4
Cryptococcal disease	31	1	0.1	0	0	10	1	10	2	11	2
Bacterial pneumonia	298	8	1.0	89	5	86	11	58	9	65	10
Other pneumonia^2^	32	1	0.1	0	0	17	2	7	1	8	1
Meningitis	38	1	0.1	10	1	9	1	12	2	7	1
Diarrhoea	31	1	0.1	1	0	12	2	11	2	7	1
HIV wasting syndrome	19	0	0.1	0	0	4	1	9	1	6	1
HIV/AIDS n.o.s.*	546	14	1.8	0	0	241	31	129	20	176	28
Other infections^3^	92	2	0.3	22	1	40	5	18	3	12	2
**Non-communicable**											
Overall	607	16	1.9	420	23	71	9	61	10	55	9
HIV related cancers^4^	49	1	0.2	0	0	17	2	14	2	18	3
Other malignancies	211	5	0.7	166	9	16	2	15	2	14	2
Cardiovascular	86	2	0.3	67	4	5	1	4	1	10	2
Hypertension/Stroke	68	2	0.2	50	3	7	1	9	1	2	0
Diabetes	20	1	0.1	15	1	0	0	4	1	1	0
Liver disease	38	1	0.1	25	1	7	1	3	0	3	0
Other^5^	134	3	0.4	97	5	19	2	12	2	7	1
**Natural causes n.o.s** *	391	10	1.3	391	21	0	0	0	0	0	0
**ALL HIV/AIDS related** **	1925	50	6.2	0	0	730	94	597	94	598	95

1 comprises 252 assaults, 73 suicides, 316 motor vehicle accidents, 143 mine accidents, 51 other accidents (such as drowning), 34 unspecified.

2 comprises 3 Cytomegalovirus, 6 nocardia, 18 interstitial pneumonitis, 3 fungal pneumonia, 2 viral pneumonia.

3 comprises 3 Candidiasis, 6 malaria, 2 aspergillus, 2 cysticercosis, 1 histoplasmosis, 1 toxoplasmosis, 29 septicaemia, 7 Hepatitis B Virus, 41 unspecified infection.

4 comprises 27 Kaposi’s sarcoma, 16 non-Hodgkin’s lymphoma, 6 lymphomas of unspecified type.

5 comprises 39 gastrointestinal disorders (such as appendicitis or gastric ulcer), 42 renal failures, 15 epilepsy or other neurological disease, 1 silicosis, 27 other respiratory diseases (such as asthma or COPD), 10 other medical causes.

* n.o.s.  =  not otherwise specified.

** includes all deaths in HIV positive people without any clearly non-HIV related cause of death.

Silicosis was identified on autopsy in 70 men. In none of these was silicosis the cause of death. In one miner, who died in 1992, the clinical cause of death was recorded on the death register as silicosis, but we have no further clinical or post-mortem information.

### HIV Programme Registration and ART

Of the 1925 men who died from HIV-related causes (Table 2), 730 (38%) died prior to ART rollout in 2003. Between 2003 and 2010, 3601 HIV-positive men were registered on the Wellness programme and 2078 started ART (Table 1). The median CD4 count at commencement of ART was 162 cells/mm^3^. There were 598 HIV-related deaths on the Wellness programme; 35% (n = 211) had never received ART. Of those HIV-related deaths that occurred on ART, 38% occurred within 6 months of starting ART. Of the 1195 who died of HIV-related causes after ART rollout, 597 (50%) had never been recorded as registered on the Wellness programme. There were no differences in the distribution of causes of death between HIV positive men who were registered on the Wellness programme and those who were not registered.

## Discussion

This study examines the population impact of a Wellness (HIV/ART) programme, rather than the outcomes of a clinic-based cohort of patients on ART. It takes into account mortality in the population as a whole, and includes those who never entered Wellness, those on ART and those who interrupt ART for a variety of reasons. This cohort of male South African platinum miners experienced a rapid increase in mortality from 1996 to 2003 due to increases in HIV and TB related mortality. Following the rollout of ART in 2003, there was an initial reduction in all-cause mortality, but a steady decrease was not sustained and HIV and TB remained the most common causes of mortality in this population. We discuss several possible explanations for these mortality trends: changing composition of the workforce, maturation of the HIV epidemic, occupational and programmatic factors.

It is well documented that there are differential rates of HIV mortality by age at infection, [21] and this may partly reflect the mortality trends observed in this study (although they persisted after adjusting for current age). The composition of this open cohort changed over the study period. From 1992 to 2004, the average age and duration of employment increased each calendar year, reflecting relatively small net turnover of employees. This was followed by periods of downsizing, such as the voluntary retrenchments in 2005 and 2009, and subsequent recruitment of large numbers of younger men. In addition to changes in the age structure, it is not known if voluntary retrenchment during these periods was associated with illness and therefore future mortality.

Interpreting the impact of ART rollout on mortality on a population level is difficult, since numerous other factors, such as changes in the population structure also impact on mortality rates over time. We do not have detailed information at an individual level for those that started and their clinical histories (eg treatment regimens, adherence, interruptions) which would be needed to fully interpret the impact of ART. The observed trends may also be a function of the natural history of HIV infection and AIDS in the population, which is dependent on, for example, HIV incidence and mortality rates by time since seroconversion. Post ART rollout in 2003 there was an initial reduction in mortality followed by an increase (spiking in 2009) and subsequent decrease in all-cause mortality. It is difficult to explain this variation, some of which may be due to chance with confidence intervals overlapping. Alternate explanations include health system failures, such as “health worker fatigue” following extensive human resources focussing on getting sick individuals on ART, but also shifting the focus away from TB services, with resultant increase in TB-specific mortality, reflected in the increasing proportion of deaths due to TB in later years.

This study has potential limitations. First, trends in all-cause mortality may be specific to this occupational cohort, but we do not believe this to be a major limiting factor because the rate of occupational injury mortality did not change significantly over the study period. [22] As part of the autopsy of cardiorespiratory organs, the presence of silicosis is recorded for compensation purposes. The 70 men with silicosis recorded on autopsy died of other causes and had probably previously worked in the gold mining industry where silicosis is much more common. Second, an occupational cohort, representing only one mine, may not be representative of the entire population, considering the “healthy worker effect” and access to relatively good quality medical services. The age-standardisation in this study used the average age distribution for the entire cohort in 10-year age groups as the standard, allowing comparison of trends across the time spectrum. Although comparison with rates standardised to the 2001 South African adult male population must be made with caution, these were very similar (8.1/1000py, compared to 9.5/1000py in 1997, and 16.3/1000py, compared to 16.7/1000py in 2004. [23]). Survival by time since HIV seroconversion in gold miners, pre-ART, has also been shown to be comparable to pre-ART survival in HIV seroconverter cohorts in developed countries. [24] A third limitation is that HIV prevalence in the population by year is not available, and we only have a single point prevalence estimate of nearly 25% in 2002. [14] We are therefore unable to estimate the proportion of the HIV-infected workforce on Wellness or on ART. We recognise that HIV may be underdiagnosed in the cohort and hence refer to HIV negative or unknown. However, in a setting with high HIV incidence, it is likely that classifying HIV status on the basis of the last negative test would result in underestimation of HIV infection at death, particularly if the test was done some time before death. This is unlikely to be a major source of bias as deaths from ‘other communicable’ diseases remain broadly stable throughout the study period and there are marked differences in causes of death between those we know to be HIV-positive and the remaining men. We did consider whether HIV infections and associated deaths were likely to be missed early in the epidemic. However, we do not believe this to be the case as mortality from other communicable diseases did not increase over that time period, whereas there was a rise in deaths from violence and injuries.

Health system, programmatic and clinical factors impact on mortality [8], [25], [26] and these would have changed over a study period which spans nearly 20 years. In order for ART to reduce mortality in a population, HIV-positive individuals need to be identified, register on an HIV care programme, start on ART at the appropriate time and continue on the treatment programme. Mine management has put initiatives in place to promote VCT services, with nearly universal coverage in later years (97% in 2010). [27] Yet, in this study, half of the HIV-related deaths in the post-ART era occurred in men who were not registered on the Wellness programme. Reasons for failure of registration on the programme may include denial of positive status, use of traditional medicines, fear of disclosure, concerns over losing employment, or language difficulties. [28] Although free access to ART is available through the mining health services, it is also possible that ART was accessed through public sector programmes by some men. Of those men who died who were registered on the programme, 35% had never been on ART. Furthermore, 38% of those who died on ART died in the early phase of treatment, indicating that they may have started treatment late. This study is not intended to evaluate the outcomes of patients on ART. A previous study in these mines showed a 71% decrease in mortality after 6–12 months of ART, compared to an historic cohort. [18] Like many other clinic-based studies, deaths among those who never registered, those who had been on treatment but were lost to follow up, and those who never received ART, were not included. In contrast, our population-based study includes all deaths and suggests that the Wellness programme is having a limited effect at a population level, with no differences in the proportion of deaths (50%) or the distribution of deaths between those in Wellness and not.

The contribution of TB-specific mortality in this cohort is striking. TB was the commonest cause of death in HIV positive miners, increasing from 28% of deaths in the pre-ART period to 41% in the post-ART period. On a population level, TB-specific mortality rates increased by an average of 14% per year over two decades. This coincided with increasing TB rates in other mine populations (TB incidence in South African gold miners doubled in HIV negative men and increased 8-fold in HIV positive men between 1991–3 and 2003–4), [16] and in the South African population generally (the number of national notified TB cases increased from 82,539 in 1992 to 396,554 in 2010). [13] Tuberculosis deaths are preventable deaths. Measures, such as screening HIV-positive individuals for TB, offering VCT to TB patients, isoniazid preventive therapy for HIV positive men, and early initiation of ART in TB/HIV patients, [8], [25] have the potential to substantially reduce mortality. Integration of HIV and TB programmes could make more efficient use of resources and help alleviate the burden of TB in people living with HIV, as well as the population as a whole. [29]

In this employment cohort, HIV and TB mortality rates remain extremely high, despite the free availability of medical care, including ART. These trends are at least in part explainable by changes in recruitment and retention in employment, and by natural changes in the stage of the HIV epidemic. However, these factors are not in the control of health services, while HIV and TB programmes and ART, are. In order to make a significant and sustained reduction in mortality in this population, expanding and integrating HIV and TB care and treatment, through programmes based on growing evidence, [8] is essential.
